# A Chemotherapy Responsive Ewing Sarcoma Case Report With a Rare FUS::FLI1 Fusion

**DOI:** 10.1155/crip/8844406

**Published:** 2025-08-15

**Authors:** Ismail M. Elbaz Younes, G. Thomas Budd, Yu-Wei Cheng

**Affiliations:** ^1^Department of Laboratory Medicine, Cleveland Clinic, Cleveland, Ohio, USA; ^2^Department of Laboratory Medicine and Pathology, Division of Hematopathology, University of Minnesota, Minneapolis, Minnesota, USA; ^3^Department of Hematology and Medical Oncology, Cleveland Clinic, Cleveland, Ohio, USA

**Keywords:** Ewing sarcoma, FLI1, FUS, gene fusion, NGS

## Abstract

Ewing sarcoma is a rare but aggressive type of cancer, primarily occurring in teenagers and young adults, characterized by having a small round cell morphology with positive diffuse membranous CD99 immunostaining of these small round blue cells. Although this cancer is often found in bones, it can also extend into the soft tissue in some cases. A gene fusion of one of the FET family RNA-binding proteins, including *EWSR1* and *FUS* genes as 5⁣′ partners, and one of the ETS family transcription factors as 3⁣′ partners, is the defining genetic characteristic of essentially all Ewing sarcoma cases. We report a case of a 42-year-old male individual with retroperitoneal Ewing sarcoma who underwent chemotherapy treatment following the biopsy diagnosis that revealed the *FUS::FLI1* fusion. To the knowledge of the authors, this is the first report of response to chemotherapy in a case of Ewing sarcoma showing a rare *FUS::FLI1* fusion.

## 1. Introduction

Ewing sarcoma is one of the differential diagnoses for a small round blue cell tumor, usually arising in the diaphysis of the long bones. Extraskeletal Ewing sarcoma occurs in around 12% of cases [1]. It is often characterized by the fusion of the FET family of RNA-binding proteins, comprising *EWSR1* and *FUS* genes as 5⁣′ partners, to one of the ETS family transcription factors as 3⁣′ partners. The most common rearrangement is the *EWSR1::FLI1* fusion that is present in 85% of cases. This chimeric fusion causes cell cycle disruption and the development of the Ewing sarcoma cells [2]. In approximately 5%–10% of cases, *EWSR1* is fused to another ETS member (*ERG*) [3]. Most of these cases are sporadic; however, germline mutations in *TP53*, *PMS2*, and *RET* have been detected in some cases [4]. The diagnostic criteria for Ewing sarcoma often involve identifying a small round cell morphology with positive membranous expression of *CD99* and the presence of a gene fusion between FET and ETS families [5]. In this report, we describe an Ewing sarcoma patient with a *FUS::FLI1* fusion treated by chemotherapy.

## 2. Case Report

A 42-year-old male individual presented to our hospital with bilious vomiting due to partial small bowel obstruction. His past medical history showed azoospermia following the repair of varicocele, which made the patient infertile. The patient was treated for the obstruction and subsequently underwent a right upper quadrant ultrasound examination for cholelithiasis, which showed a 5 cm retroperitoneal mass. A PET-CT scan showed the same lesion with increased uptake in the retroperitoneum between the aorta and inferior vena cava. The mass measured 4.2 × 5.5 × 5.8 cm, and no lymphadenopathy was identified. The patient recovered from the small bowel obstruction and presented to our hospital for the treatment of the mass.

**Figure 1:**
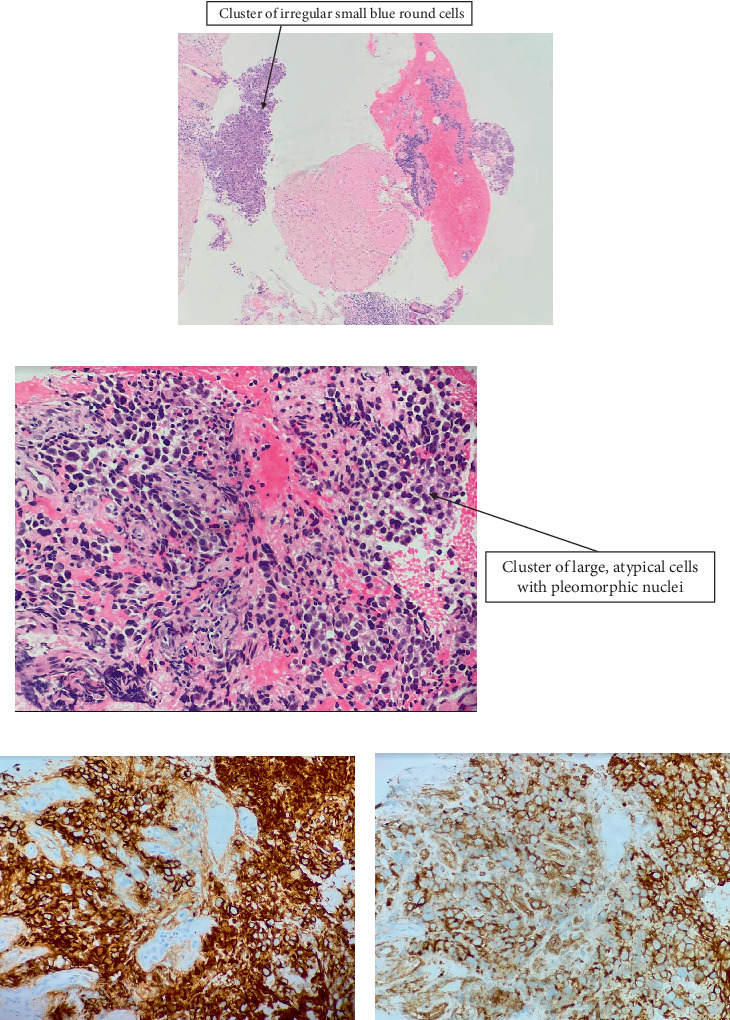
(a) 40x magnification showing small fragments of a cellular small round blue cell neoplasm with crush artifact and normal small intestine. (b) 200x magnification showing higher power of the small clusters of cells highlighting the irregular hyperchromatic nuclei with “smudgy” chromatin and indistinct nucleoli with scant to no cytoplasm. (c) 200x showing expression of neoplastic cells of cytokeratin AE1/AE3. (d) 200x showing expression of neoplastic cells by CD99.

A CT scan-guided percutaneous needle biopsy of the lesion showed a poorly differentiated population of cells with round cell features, and no necrosis was identified (Figure 1a). FDG PET scan performed prior to pathologic diagnosis showed no distant metastases. CT scans of the chest, abdomen, and pelvis performed prior to the initiation of therapy showed slight progression of the retroperitoneal mass, but no distant metastases. Two months after the neoplastic lesion was detected, the patient received the first cycle of chemotherapy with vincristine, doxorubicin, and cyclophosphamide (VDC). He tolerated treatment well in the immediate posttreatment period but was hospitalized for a perianal abscess, which was treated by incision and drainage. The patient received the second cycle of chemotherapy with ifosfamide and etoposide (IE). Three months after the first cycle of chemotherapy, the follow-up CT scan showed a reduction of the lesion size from 5.9 to 4 cm. The patient received treatment cycles 3–6 and responded well, except that he developed *Clostridioides difficile* colitis for which he received vancomycin treatment. The seventh cycle of chemotherapy with VDC was complicated by a prolonged hospitalization for neutropenic fever and acute kidney injury attributable to IV vancomycin, sepsis, and prior ifosfamide. His renal function slowly improved at home, but he had an episode of atrial fibrillation with rapid ventricular response while hospitalized, resulting in cardioversion. The patient received an additional seven cycles of VDC alternating with IE treatment every 2–3 weeks at the tolerated doses as described in the literature, thus completing a total of 14 cycles of chemotherapy [6, 7]. Ten months after the first cycle of chemotherapy, the patient underwent cholecystectomy as well as exploratory laparotomy. The excised retroperitoneal mass showed a residual size of 3.5 cm in greatest dimension. Pathologic review of the residual 3.5 cm mass showed 95% necrosis and 5% viable tumor cells, without other treatment-related lesions. Overall, the patient did well most of the time, with no new symptoms suggesting recurrence. His bouts of small bowel obstruction from adhesions had decreased in frequency and were managed at home. The patient remains free of recurrence 5 years after the initial diagnosis.

**Figure 2:**
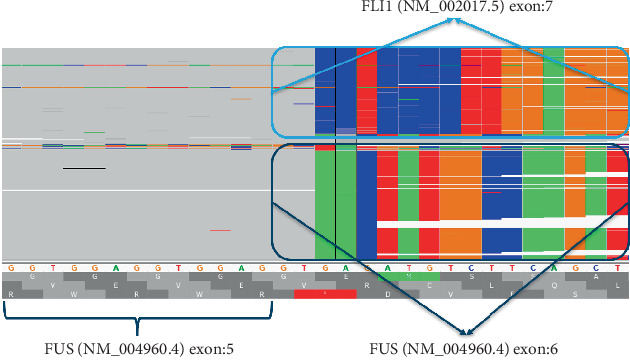
The identified FUS::FLI1 in-frame fusion showing relatively abundant transcription. The fusion junction is at chr16:31195717–chr11:128677075 (hg19).

Grossly, the pretreatment biopsy specimen showed a tumor consisting of multiple irregularly shaped fragments of pink-red tissue aggregating to 0.7 × 0.7 × 0.1 cm. Histologic examination revealed sheets of large pleomorphic atypical cells with prominent nucleoli and ranging from spindled shaped cells to round cells (Figure 1b). The differential diagnosis for this pattern was a poorly differentiated carcinoma with focal expression of neuroendocrine markers (synaptophysin, CD56), an Ewing sarcoma, and a desmoplastic small round cell tumor. Immunohistochemical studies were done and showed strong expression of AE1/AE3 keratin (Figure 1c). There was also positive membranous staining for CD99 (Figure 1d), CAM5.2, CD56, and synaptophysin. However, they were negative for OCT 3/4, myogenin, DUX4, desmin, CD43, D2-40, AFP, Beta-hCG, and DOG-1. PLAP showed patchy staining of uncertain significance. A next generation sequencing of the sarcoma fusion gene panel was performed at the Cleveland Clinic and identified a relatively abundant *FUS::FLI1* gene fusion (Figure 2) [8] . This chimeric transcript fused *FUS* Exon 5 (NM_004960.4) to *FLI1* Exon 7 (NM_002017.5) (also known as *FLI1* Exon 8 under transcript NM_001167681.2).

## 3. Discussion

The most frequent gene fusion identified in Ewing sarcoma is *EWSR1::FLI1* fusion, and the second most common fusion is *EWSR1::ERG*. We present a case of Ewing sarcoma with an unusual histologic picture coupled with a very rare *FUS::FLI1* fusion. The patient has responded well to the treatment, with successful follow-up visits over a 54-month period at the time of this manuscript's submission. Indeed, the degree of tumor necrosis following VDC chemotherapy is predictive of the treatment response in cases of Ewing sarcoma, osteosarcoma, and desmoplastic small round cell tumor [9–11]. Patients who exhibit high tumor necrosis (for instance, > 90%) postchemotherapy demonstrate a significantly reduced risk of disease recurrence in comparison to those with less necrosis [12]. In the case of our patient, the excised retroperitoneal mass post-VDC therapy displayed 95% necrosis, consistently indicating the successful treatment outcome. To the best of our knowledge, this rare fusion has been previously reported in two cases of Ewing sarcoma [13, 14] and one case of pediatric acute myeloid leukemia [15]. However, none of these prior publications provided any information regarding treatment. Given its rarity and that none of the previous cases had therapy opportunities to demonstrate the responses, we are reporting this case to the literature.

While Ewing sarcoma is the most common type of undifferentiated small round cell sarcoma, other types of undifferentiated small round cell sarcomas (shown in Table 1) with subtle morphologic feature differences may cause a diagnostic challenge to the practicing pathologist. The differential diagnoses for Ewing sarcoma include atypical Ewing sarcoma and adamantinoma-like Ewing sarcoma (ALES). Atypical Ewing sarcoma involves a large tumor cell with prominent nucleoli [27]. ALES involves a diffuse pattern of pancytokeratin expression, the presence of high-molecular weight keratin, positive stains of p40 and p63, and shows squamous differentiation [28]. Ewing sarcoma typically lacks epithelial features (e.g., tumor cells do not express keratin AE1/AE3), or these features only show in a subset of cases [29].

In addition to ALES, small cell synovial sarcoma (also known as round cell synovial sarcoma) can also be included in the differential diagnosis for Ewing sarcoma. Small cell synovial sarcoma frequently shows expression of epithelial membrane antigen (EMA), although the staining may be focal and less intense than that of Ewing sarcoma. While the cytokeratin expression in small cell synovial sarcoma can be variable, its presence can still be helpful in a synovial sarcoma diagnosis when interpreted in conjunction with other markers like CD99 and TLE1 [30, 31]. Overall, it is important to incorporate molecular genetic methods to differentiate between Ewing sarcoma and other lesions that might share the histologic features. In conclusion, we have presented a case of retroperitoneal Ewing sarcoma characterized by an uncommon FUS::FLI1 fusion. As far as we are aware, this is the first documented report of a successful chemotherapy response in an Ewing sarcoma patient with this specific gene fusion.

## Figures and Tables

**Table 1:** Other types of undifferentiated small round cell sarcomas.

**Entity**	**Genetic workup**	**Keratin expression**
Round cell sarcomas with *EWSR1*-non-ETS fusions	ETS*. EWSR1-NFATC2*, *FUS-NFATC2*, and *EWSR1-PATZ1* fusions [16–18]	Focal AE1/AE3 dot-like staining [19]
CIC-rearranged sarcoma (*CIC*-*DUX4* sarcoma)	*CIC-DUX4* in 95% of cases [20] Non-*DUX4* partners such as *NUTM1*, *NUTM2A*, *LEUTX*, and *FOXO4* [21–24]	Focal staining of AE1/AE3 in a small subset of cases [25]
Sarcoma with *BCOR* genetic alterations	*BCOR*-*CCNB3* and *BCOR*-ITD [26]	Negative for AE1/AE3

## Data Availability

The data used to support the findings of this study are included within the article. Other details may be available from the corresponding author upon request. Data will be shared in accordance with institutional guidelines and after obtaining necessary permissions.
